# Road Traffic Accidents among Patients Visiting Department of Emergency of a Tertiary Care Centre: A Descriptive Cross-sectional Study

**DOI:** 10.31729/jnma.8135

**Published:** 2023-04-30

**Authors:** Shanta Sharma, Indra Dhakal, Madhav Bhusal, Nilam Adhikari, Subhash Bhusal, Tekraj Upadhaya, Ishan Subedi, Anil Tiwari, Priya Verma, Bikash Dhital, Neha Pandey, Anup Panthi

**Affiliations:** 1 Department of Community Medicine, Devdaha Medical College and Research Institute, Bhaluhi, Rupandehi, Nepal; 2 Department of Orthopedics and Trauma, Lumbini Provincial Hospital, Butwal, Rupandehi, Nepal; 3 Department of Public Health and Community Medicine, B.P. Koirala Institute of Health Sciences, Dharan, Sunsari, Nepal; 4 Department of Dentistry, Lumbini Provincial Hospital, Butwal, Rupandehi, Nepal; 5 Department of Community Medicine, National Medical College, Birgunj, Parsa, Nepal; 6 Department of Community Medicine, Maharajgunj Medical Campus, Maharajgunj, Kathmandu, Nepal

**Keywords:** *emergencies*, *prevalence*, *traffic accidents*

## Abstract

**Introduction:**

Road Traffic Accidents, are one of the major neglected global health burdens which are predicted to be the 7^th^ leading cause of global deaths by 2030 as per the World Health Organization hence, seem to be one of the major global threats in near future. Most road traffic accidents affect the most vulnerable age groups in developing countries. The aim of this study was to find out the prevalence of road traffic accidents among patients visiting the Department of Emergency of a tertiary care centre.

**Methods:**

A descriptive cross-sectional study was conducted among patients visiting the department of emergency of a tertiary care centre from 16 September 2022 to 15 October 2022. Ethical approval was obtained from the Institutional Review Committee (Reference number: IRC-DMCRI: 307/079/080). All the road traffic accidents cases recorded in the Emergency Department from 14 April 2021 to 13 April 2022 were taken. Convenience sampling was used. Point estimate and 95% confidence interval were calculated.

**Results:**

Among 29735 patients, the prevalence of road traffic accidents was 1340 (4.50%) (4.26-4.74, 95% Confidence Interval). Among these, 1037 (77.4%) were male and 303 (22.6%) were female. Road traffic accidents among two-wheelers were 1065 (79.48%) followed by pedestrian 703 (52.46%). Mangsir witness the higher number of cases, 137 (13.90%) followed by Kartik, 170 (12.69%).

**Conclusions:**

The prevalence of road traffic accidents was similar to other studies done in similar settings. In our study, young people of highly productive and active age groups were the most common victims.

## INTRODUCTION

Road traffic injuries (RTA) stand as the 9^th^ leading cause of death globally in 2012 while it is predicted to be the 7^th^ leading cause of global deaths by 2030.^1^ The majority of road accident deaths are currently among "vulnerable road users", pedestrians, pedal cyclists and motorcyclists.^2^

Road traffic injuries are a major but neglected global public health problem, requiring concerted efforts for effective and sustainable prevention. The mortality rate in the South-East Asia Region is 18.5 per 100,000 population and one-third of those deaths involve motorized 2-3 wheelers.^3^ In Nepal, a total of 95,902 crashes, 100,499 injuries and 14,512 deaths were recorded by the traffic police over the 12 years, 2001-2013.^4^ In 2020, Nepal reported 15,554 road traffic accidents. Among them, around 29.6% were severely injured while 14.5% reached deaths.^5^

The aim of this study was to find out the prevalence of road traffic accidents among patients visiting the Department of Emergency of a tertiary care centre.

## METHODS

A descriptive cross-sectional study was conducted among patients visiting the Department of Emergency of a tertiary care centre from 16 September 2022 to 15 October 2022 after obtaining ethical approval from the Institutional Review Committee (Reference number: IRC-DMCRI: 307/079/080). All the patients visiting the Department of Emergency with complete hospital data were included in the study. Any injury on the road without the involvement of a vehicle (e.g. person slipping and falling on the road and sustaining injury) and accidents with bicycles was excluded from the study. Missing data were also excluded from the study. A convenience sampling method was used. All the RTA cases recorded in the Emergency Department from 14 April 2021 to 13 April 2022 were taken. Sample size was calculated by using the following formula:


$$\begin{array}{ll} \text{n} & ={\text{Z}}^{2}\times \frac{\text{p}\times \text{q}}{{\text{e}}^{\text{2}}}\\ & =1.96^{2}\times \frac{0.50\times 0.50}{0.01^{2}}\\ & =9604 \end{array}$$

Where,

n = minimum required sample size
Z = 1.96 at 95% Confidence Interval (CI)
p = prevalence taken as 50% for maximum sample size
q = 1-p
e = margin of error, 1%

The calculated sample size was 9604. Doubling the sample size, the total sample size was 19,208. However, a total of 29,735 patients were taken. The data was collected retrospectively from the police case report recorded at the Department of Emergency.

Data were entered in Microsoft Excel Version 2010 and analyzed using IBM SPSS 20.0. Point estimate and 95% CI were calculated.

## RESULTS

Among 29735 patients, the prevalence of road traffic accidents was 1340 (4.50%) (4.26-4.74, 95% CI). Among these, 1037 (77.4%) were male and 303 (22.6%) were female. Out of them, the total mortality was found to be 106 (7.91%). Most victims 1041 (77.69%) fall in the 16-45 years age group and 910 (67.9%) of victims belonged to the Rupandehi district itself (Table 1).

**Table 1:** Socio-demographic characteristics (n = 1340).

Characteristics		n (%)
Sex	Female	303 (22.6)
	Male	1037 (77.4)
Age of victims	< 15	114 (8.51)
	16-30	681 (50.82)
	31-45	360 (26.87)
	46-60	127 (9.48)
	> 60	58 (4.33)
District of the victims	Rupandehi	910 (67.91)
	Nawalparasi	84 (6.27)
	Kapilvastu	78 (5.82)
	Palpa	70 (5.22)
	Gulmi	59 (4.40)
	Arghakhanchi	47 (3.51)
	Dang	24 (1.79)
	Pyuthan	14 (1.04)
	Others	54 (4.03)

**Figure 1:**
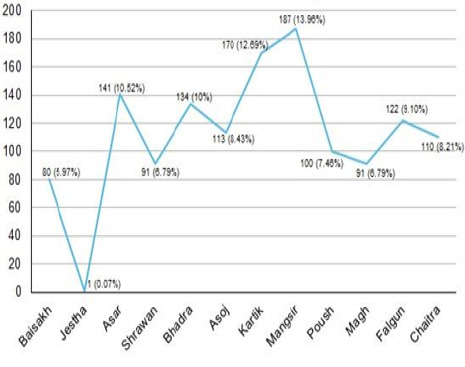
Road traffic accidents on a monthly basis (n= 1340).

In Mangsir 137 (13.90%) witnessed the highest number of road traffic accident victims (Figure 1).

**Figure 2:**
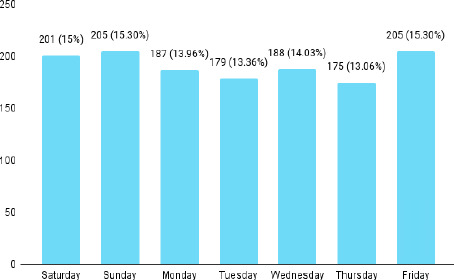
Road traffic accidents in a week (n= 1340).

The prevalence of RTA was 205 (15.30%) on Friday and Sunday followed by 201 (15%) on Saturday (Figure 2).

Two-wheeler 1065 (79.48%) and pedestrians 703 (52.46%) were the most common vehicle and most common victims involved in RTA respectively (Table 2).

**Table 2:** Vehicle type and victim group (n= 1340).

Characteristics	n (%)	
Vehicle type	Two wheeler	1065 (79.48)
	Light motor vehicle	181 (13.51)
	Heavy motor vehicle	94 (7.01)
Victim group	Pedestrian	703 (52.46)
	Driver	487 (36.34)
	Pillion rider	112 (8.36)
	Passenger	38 (2.84)

Limbs 1039 (77.53%) were the most common site injured with blunt injury being the most common type of injury 967 (72.16%) while 138 (10.30%) of the victims presented with loss of consciousness (Table 3).

**Table 3:** Medical aspects of the accident victims (n= 1340).

Characteristics	n (%)	
Site of body injured	Limbs	1039 (77.53)
	Head and neck	288 (21.49)
	Face region	202 (15.07)
	Abdomen and pelvis	92 (6.87)
	Thorax	72 (5.37)
Types of injury	Blunt Injury	967 (72.16)
	Laceration/abrasion	315 (23.51)
	Multiple/mixed Injuries	30 (2.24)
	Fracture	28 (2.09)
Loss of consciousness	Present	138 (10.30)

## DISCUSSION

The prevalence of road traffic accidents was 1340 (4.50%) which is similar to the findings done in similar research which showed RTA cases 6.66% of the total Emergency cases.^6^ Most of the victims attending the hospital were male comprising 1037 (77.4%) of the total victims which is consistent with other research conducted in South Asia in 2003 among 726 victims in Jawaharlal Institute of Post Graduate Medical Education and Research Hospital, Pondicherry which showed 83% of male victims.^7^ This can be attributed to the higher number of male drivers including reckless driving among male drivers. The highest number of victims i.e. 681 (50.82%) was found in the age group between 16-30 years of age followed by the 31-45 years age group i.e. 360 (26.87%). This comprises about 77.69% of the total victims of road traffic accidents in the most productive age groups of 16-45 years which has similar findings in similar research i.e. 69.2% of the victims fall in the 20-49 age groups.7 An average of 122 RTA victims attended the hospital monthly which shows the burden of hospitals for traffic injuries and the need for dedicated trauma care set up in the area. Due to the Government imposed nationwide lockdown, Jestha showed no RTA victims in the hospital which is in contrast with the findings of similar research on Road traffic injuries in Nepal during the COVID-19 lockdown (24 March to 14 June 2020) which showed 22.2 injuries daily nationwide.8 As our data relies on the victims visiting the hospital only, the findings seem inconsistent with other research. Friday 205 (15.30%), Saturday 201 (15.00%) and Sunday 205 (15.30%) witnessed the highest number of admission of victims in the hospital which is similar to another research.^9^ Considering the highest number of victims from the Rupandehi district 910 (67.91%) itself, the visit day of victims and the accident day can be correlated and hence, the highest accidents seem to occur mostly at the weekends which can be attributed mostly to vacation travelling, late night parties and drunk and drive on weekends.

In our study, motorized two-wheelers were the most affected victims 1065 (79.48%) which is similar to the findings (approximately 70%) conducted in similar research on Road Traffic accidents at Trauma centre MDM Hospital Jodhpur, an article which was published in International Journal Of Scientific Research on June 2022 issue.^9^ Pedestrians were the most common victims 703 (52.46%) followed by drivers 487 (36.34%) attending the hospital. This can be attributed to the poor road condition of the area and the need for footpaths along with the safety of the pedestrians. Similar results were reported in An epidemiological study of road traffic accident cases attending the emergency department of a teaching hospital; the most common victims being pedestrians (55.64%) followed by driver/front rider (25.36%).^6^ Our research suggested that the most common injury sustained by the victims were in the limbs 1039 (77.53%) followed by head and neck injuries 288 (21.49%). This data is in contrast with similar research conducted in Western Nepal among Road traffic trauma patients who were brought to the Emergency Department of Manipal Teaching Hospital, Pokhara, Nepal, during 1 year, i.e. 1 January 2003 to 31 December 2003, which reveals head and face injuries and injuries to the lower limbs comprised 58.1% and 50.7% of all injuries respectively.^10^ But, another research revealed that the limbs (63.1%) and face (17.5%) were the most commonly affected areas to suffer the injuries.^7^ This can be due to various factors of the accidents like commonest motorized two-wheelers injuries which sustain injuries over limbs and pedestrians who were the most common victims sustaining force with the limbs.

In our study, 967 (72.16%) of the victims were found to have blunt injury followed by lacerations/ abrasions 315 (23.51%), fractures 28 (2.09%) and multiple injuries 30 (2.24%). Another similar research reported around 30% of victims with fractures.^7^ In our study, 138 (10.30)% of victims among 1340 victims reached the hospital with loss of consciousness. This is in consideration that the loss of consciousness, if present, should have been documented in the Police Case Report. But, as per Nepal Police records documented in the Police Case Report for 10,178 road crashes that occurred in the fiscal year 2016/17, 4250 victims with serious injuries (who are unconscious after the crash) makes up around 41% of the crashes.^5^

As the data is taken from a single hospital in the province, these data cannot be generalized to the whole population. It does not represent the burden of the RTAs in the country. As our study is taken from hospital records, many epidemiological factors could not be incorporated. We could not study the accident time and visit time and hence calculate the time elapsed since the crash to study the golden hour time frame and its importance in saving the part of the body, disability limitation and saving the life itself. The financial burden and follow-up treatment of RTAs could not be studied due to the absence of information and the limitation of our study.

## CONCLUSIONS

The prevalence of road traffic accidents was similar to other studies done in similar settings. Our study revealed that the majority of the RTA victims were male, mostly on two-wheelers, of the most active and productive age group (16-45 years), and pedestrians and accounted for about 8% of deaths among the victims. The most common injuries were found on limbs followed by head and neck, both of which can lead to serious disability and even fatalities; which strongly suggests the need for a dedicated trauma care centre in the area.
